# Bloodstream Infections in the Neonatal Intensive Care Unit: A Systematic Review of the Literature

**DOI:** 10.7759/cureus.68057

**Published:** 2024-08-28

**Authors:** Georgia Vogiantzi, Dimitra Metallinou, Maria Tigka, Anna Deltsidou, Christina I Nanou

**Affiliations:** 1 Department of Midwifery, Faculty of Health and Care Sciences, University of West Attica, Athens, GRC; 2 Department of Midwifery, General and Maternity Hospital "Helena Venizelou", Athens, GRC

**Keywords:** antibiotic therapy, neonatal sepsis, susceptibility, resistance, antibiotics, antimicrobial resistance, pathogens, neonate, neonatal intensive care unit, bloodstream infections

## Abstract

Bloodstream infections represent a significant concern in neonatal intensive care units (NICUs), constituting a leading cause of morbidity and mortality among neonates. This study aimed to elucidate the etiology, prevalence, and antimicrobial resistance patterns of bloodstream infections in NICU settings. A systematic review was conducted according to the Preferred Reporting Items for Systematic Reviews and Meta-Analyses (PRISMA) statement guidelines using the PubMed database to source relevant studies published between 2019 and 2023. Keywords related to bloodstream infections, neonates in NICUs, pathogens, resistance, and susceptibility were employed. Out of the 73 identified articles, eight met the inclusion criteria. Findings revealed a predominance of late-onset sepsis in hospitalized neonates, with *Escherichia coli*, *Klebsiella pneumoniae*, coagulase-negative staphylococci, Group B *Streptococcus*, *Acinetobacter* species, *Serratia marcescens*, *Staphylococcus aureus*, and *Enterobacter cloacae* being the most commonly isolated pathogens. Antimicrobial susceptibility profiles demonstrated resistance among bacteria to ampicillin, gentamicin, and penicillin, while fungi exhibited resistance to amphotericin B, fluconazole, flucytosine, itraconazole, and voriconazole. These findings underscore the persistent challenge of bloodstream infections in the NICUs, particularly late-onset sepsis, emphasizing the importance of early detection and appropriate antimicrobial therapy in neonatal care management.

## Introduction and background

Bloodstream infections represent the third leading cause of morbidity and mortality among neonates globally [1]. This underscores the critical need for in-depth research into bloodstream infections within this vulnerable population. Previous evidence reports an incidence of neonatal sepsis at 2,824 cases per 100,000 live births with a mortality rate of 17.6% [2]. Notably, mortality rates inversely correlate with age, as premature neonates exhibit higher mortality rates compared to the full-term ones [3].

Sepsis, clinically recognized as a manifestation of primary bacteremia without an identifiable source of infection [4], is subdivided into early- and late-onset sepsis. In neonates admitted to the neonatal intensive care unit (NICU), early-onset sepsis is defined as infection manifesting within 72 hours of birth [5], frequently stemming from an ascending infection subsequent to membrane rupture during pregnancy, and involving maternal vaginal microorganisms and exposure to potential pathogens within the birth canal. Conversely, late-onset neonatal sepsis is frequently linked to microorganisms colonizing the hospital environment [6]. Additionally, neonates hospitalized in a NICU are particularly susceptible to late sepsis due to factors such as prolonged use of central lines, parenteral nutrition, and delayed initiation of enteral feeding [7].

The gold standard for the diagnosis of bloodstream infection is the blood culture [8]. Complementary laboratory diagnostics include measurements of C-reactive protein (CRP) and procalcitonin levels, which aid in diagnosis [8,9]. Specifically, CRP measurements within the first 48 hours post-symptom onset enhance test sensitivity, with normal CRP values indicating a 99% negative predictive value for infection [10,11]. However, the interpretation of elevated CRP levels in early sepsis can be complicated by preexisting conditions such as premature rupture of the fetal membranes, antenatal steroid administration, and fetal distress, which may elevate CRP levels [12,13]. Procalcitonin, released from tissues in response to infection, rises more rapidly than CRP, making it an effective early marker for sepsis detection [8].

Regarding treatment, the World Health Organization (2022) recommends the administration of ampicillin or penicillin in conjunction with gentamicin for a minimum duration of 10 days as a first-line treatment approach for culture-positive neonatal sepsis [14], with antibiotic regimens adjusted based on culture results and the susceptibility profile of the isolated pathogen [4]. This necessitates ongoing research into the resistance patterns of pathogens to optimize therapeutic strategies for sepsis and its associated outcomes.

The aim of this systematic review was to investigate the prevalence, pathogen spectrum, and antimicrobial resistance patterns of bloodstream infections in hospitalized neonates.

## Review

Materials and methods

**Figure 1:**
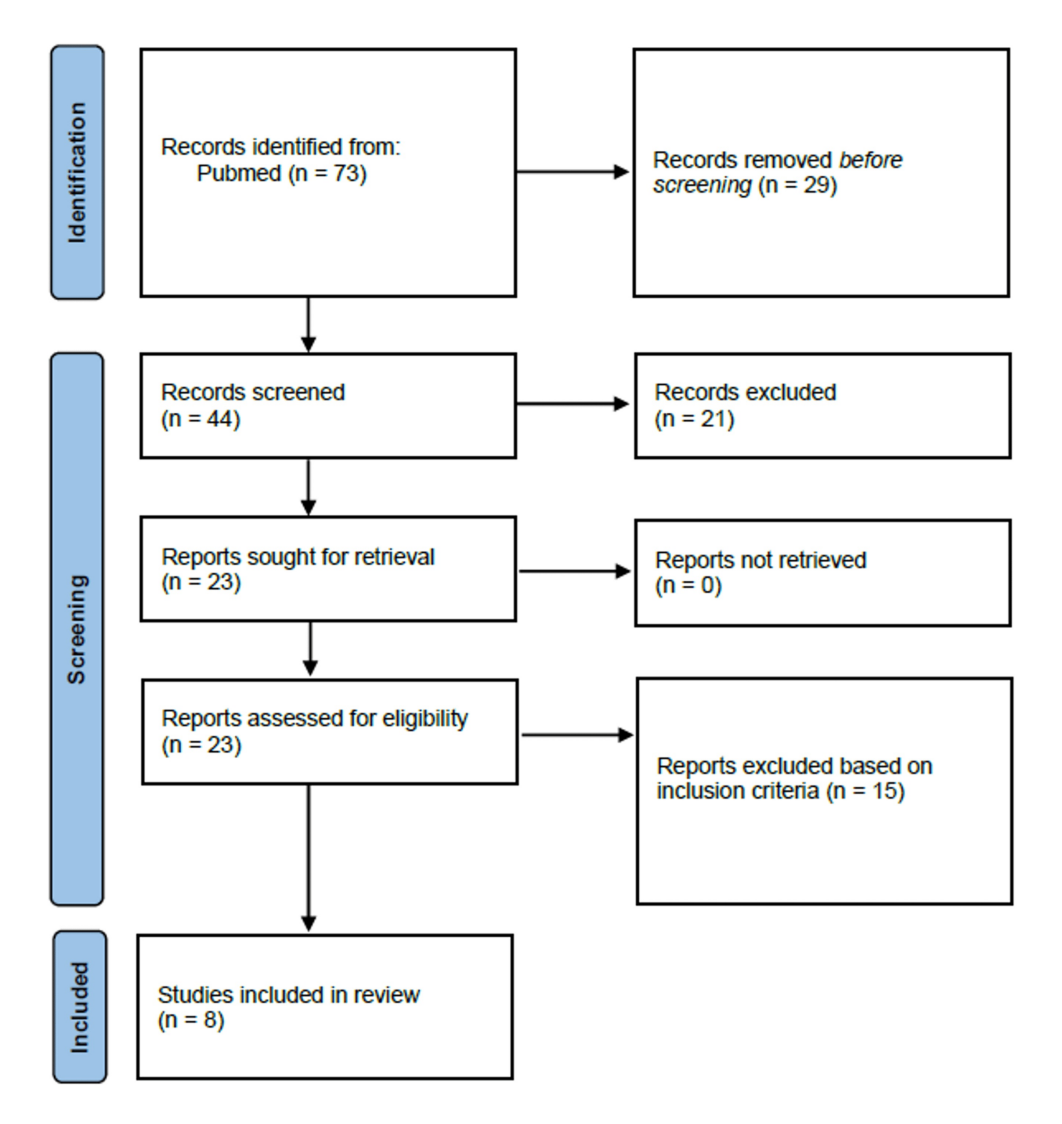
PRISMA flow diagram PRISMA: Preferred Reporting Items for Systematic Reviews and Meta-Analyses

A comprehensive literature search was conducted according to the Preferred Reporting Items for Systematic Reviews and Meta-Analyses (PRISMA) statement guidelines using the PubMed database to source relevant studies published between 2019 and 2023. The search strategy employed specific keywords and Boolean operators including the following: ("bloodstream infections" OR "BSI") AND ("neonates" OR "newborns" OR "infants") AND ("neonatal intensive care unit" OR "NICU") AND ("neonatal sepsis") AND ("pathogens" OR "microorganisms") AND ("susceptibility" OR "vulnerability" OR "sensitivity") AND ("antimicrobial resistance") AND ("antibiotics" OR "antibiotic therapy"). Initially, this strategy yielded 73 articles. Subsequent screening involved a rigorous selection process where 29 articles were excluded based on title relevance and an additional 21 were excluded after abstract review, primarily due to irrelevance to the research focus or inadequate data. The full texts of the remaining 23 articles were meticulously examined against specific inclusion and exclusion criteria. The criteria stipulated that the studies should involve neonates hospitalized in NICUs, be published in English from 2019 to 2023 and in peer-reviewed journals, and adopt retrospective, cross-sectional, or cohort study designs. Exclusion criteria were non-English publications, publication outside the specified date range, studies focusing on older children, unavailability of full text, and systematic reviews or meta-analyses (Figure 1).

**Figure 2:**
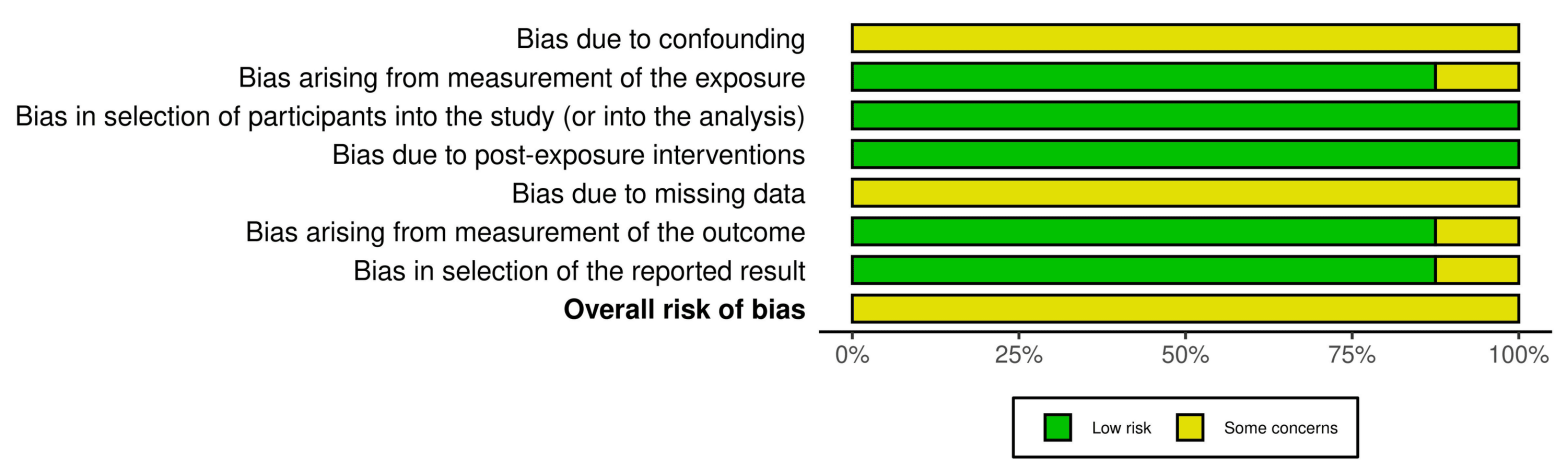
Overall risk of bias of the included studies

All eight studies were evaluated using the risk of bias in non-randomized follow-up studies of exposure effects (ROBINS-E) tool [15], and each was found to have a moderate overall risk of bias. Detailed evaluations for each study are available in Table 1. This assessment covered potential biases in seven bias domains. The overall risk of bias evaluation, visualized with risk-of-bias visualization (robvis) [16], indicated that all studies showed some concerns (Figure 2), mainly including potential confounding and missing data. These findings underscore common challenges in observational research, especially in retrospective studies and those spanning extended time frames.

**Table 1:** Detailed assessment of risk of bias for each study, conducted using the ROBINS-E tool NICU: neonatal intensive care unit; ROBINS-E: risk of bias in non-randomized follow-up studies of exposure effects

Sana et al., 2019	
Bias due to confounding	Risk of bias: Moderate.
	Justification: The study mentions that neonatal risk factors such as low birth weight and prematurity were analyzed, which indicates some level of control for confounding. However, the study does not appear to include a comprehensive list of potential confounders or describe how they were controlled in the analysis.
Bias in the selection of participants	Risk: Low.
	Justification: The study was descriptive and cross-sectional, including all neonates admitted to the NICU during the study period who had blood cultures taken. This reduces the risk of selection bias. The inclusion criteria seem broad and representative of the target population.
Bias in the classification of exposures	Risk: Low.
	Justification: Accurate classification of infections and susceptibility profiles reduces the likelihood of classification bias.
Bias due to deviations from intended exposures	Risk: Low.
	Justification: There are no deviations from the intended exposures as the study did not manipulate the exposures.
Bias due to missing data	Risk: Moderate.
	Justification: The study does not report any missing data or how missing data was handled. Missing data could introduce bias, particularly if records were incomplete or missing.
Bias in the measurement of outcomes	Risk: Moderate.
	Justification: The outcomes (bloodstream infections and their antibiotic susceptibility profiles) were measured using standard laboratory procedures, reducing the risk of bias. However, the study relied on clinical signs for diagnosing sepsis, which could be subjective.
Bias in the selection of the reported result	Risk: Moderate.
	Justification: The study appears to report all relevant outcomes related to the objective, but it is not clear if all collected data were reported. There is a potential for selective reporting if not all outcomes were presented.
Overall risk of bias	The overall risk of bias in this study appears to be Moderate. The study was well-conducted in terms of selecting participants and measuring outcomes, but there are concerns regarding confounding, potential missing data, and the subjectivity of diagnosing sepsis.
Li et al., 2019	
Bias due to confounding	Risk: Moderate
	Justification: While confounders are considered, there is no detailed explanation of how all potential confounders were handled or adjusted for in the analysis, particularly for factors that could influence both the exposure (e.g., types of bacteria) and the outcomes.
Bias in the selection of participants	Risk: Low.
	Justification: Clear inclusion criteria reduce the risk of selection bias.
Bias in the classification of exposures	Risk: Low.
	Justification: The classification appears to be clear and based on well-established criteria.
Bias due to deviations from intended exposures	Risk: Low.
	Justification: The study design minimizes concerns about deviations from intended exposures.
Bias due to missing data	Risk: Moderate.
	Justification: A considerable number of cases were excluded due to incomplete data. This could affect the study’s findings if the missing data were not random. The study does not provide details on how missing data were addressed or whether sensitivity analyses were performed to assess the impact of this missing data.
Bias in the measurement of outcomes	Risk: Low.
	Justification: Outcomes were measured using standardized clinical and laboratory procedures.
Bias in the selection of the reported result	Risk: Low.
	Justification: The study reports on a wide range of outcomes, including clinical characteristics, risk factors, and antibiotic susceptibility patterns. There is no indication of selective reporting.
Overall risk of bias	The overall risk of bias in this study is likely Moderate. While it provides valuable insights into neonatal sepsis, particularly in the context of pathogen distribution and antibiotic resistance, the issues related to missing data and potential residual confounding are notable limitations.
Mintz et al., 2020	
Bias due to confounding	Risk: Moderate.
	Justification: The study addresses confounders in a general sense but does not provide a detailed strategy for adjusting for potential confounding factors.
Bias in the selection of participants	Risk: Low.
	Justification: Participants were selected based on clear criteria (neonates with bacteremia), reducing the likelihood of selection bias. The selection of participants is well-defined and appropriate for the research question.
Bias in the classification of exposures	Risk: Low.
	Justification: The classification of exposure (bacteremia type and pathogens) is clear and follows established criteria.
Bias due to deviations from intended exposures	Risk: Low.
	Justification: As an observational study, there are minimal concerns regarding deviations from intended exposures.
Bias due to missing data	Risk: Moderate.
	Justification: The study does not provide detailed information on how missing data were handled. Given the long study period, it is likely that some data points were incomplete or missing, but this is not explicitly addressed.
Bias in the measurement of outcomes	Risk: Low.
	Justification: The outcomes are measured using standard laboratory techniques, which should provide accurate and reliable data. However, the study does not discuss potential variations in measurement techniques over the 10-year period.
Bias in the selection of the reported result	Risk: Low.
	Justification: The study reports on a wide range of outcomes related to bacterial prevalence and antibiotic resistance patterns. There is no indication of selective reporting.
Overall risk of bias	The overall risk of bias in this study is likely Moderate. While the study is well-conducted and provides valuable insights into the changing epidemiology of neonatal bacteremia, some areas, particularly regarding confounding factors and handling of missing data, present moderate risks of bias.
Liu et al., 2021	
Bias due to confounding	Risk: Moderate.
	Justification: While the study considers important confounders, the lack of a detailed strategy for adjusting these factors suggests a moderate risk of residual confounding.
Bias in the selection of participants	Risk: Moderate.
	Justification: The selection of participants is appropriate, but the exclusion of neonates without culture results could lead to selection bias.
Bias in the classification of exposures	Risk: Low.
	Justification: The classification of exposure is clear and based on standard criteria.
Bias due to deviations from intended exposures	Risk: Low.
	Justification: The study is observational and does not involve any interventions that could lead to deviations from intended exposures.
Bias due to missing data	Risk: Moderate.
	Justification: The study does not provide extensive details on how missing data were handled, but it excludes a significant number of potential contaminants from the analysis, which may introduce bias. The impact of these exclusions on the study's findings is not fully explored.
Bias in the measurement of outcomes	Risk: Low.
	Justification: Outcomes are measured using standardized microbiological techniques, including blood culture identification and antimicrobial susceptibility testing. The study uses consistent methods across multiple NICUs, though it does not discuss potential variability in measurement practices over time.
Bias in the selection of the reported result	Risk: Low.
	Justification: The study appears to comprehensively report all relevant findings without evidence of selective reporting.
Overall risk of bias	The overall risk of bias in this study is likely Moderate. The study is well-conducted and provides valuable insights into neonatal sepsis and antimicrobial resistance in China. However, the lack of detailed adjustments for confounding factors, the handling of missing data, and the potential for variability in measurement practices contribute to a moderate risk of bias.
Oo et al., 2021	
Bias due to confounding	Risk: Moderate
	Justification: While the study addresses some confounders, the potential for residual confounding remains due to limited adjustment strategies.
Bias in the selection of participants	Risk: Low.
	Justification: The selection process is clear, but excluding neonates without culture results could introduce bias.
Bias in the classification of exposures	Risk: Low.
	Justification: The classification of exposures appears reliable, but the potential for misclassification of culture-negative cases is a minor concern.
Bias due to deviations from intended exposures	Risk: Low.
	Justification: This is an observational study that does not involve any interventions that could lead to deviations from intended exposures.
Bias due to missing data	Risk: Moderate.
	Justification: The lack of information on missing data and its handling introduces some uncertainty.
Bias in the measurement of outcomes	Risk: Low.
	Justification: While the outcome measurements are likely accurate, the lack of discussion on potential variations over time is a concern.
Bias in the selection of the reported result	Risk: Low.
	Justification: The study appears to report all relevant results comprehensively.
Overall risk of bias	The overall risk of bias in this study is likely Moderate. The study provides important insights into neonatal sepsis and antibiotic resistance in Myanmar, but the lack of detailed adjustments for confounding factors, handling of missing data, and potential variations in measurement practices are notable limitations.
Salah et al., 2021	
Bias due to confounding	Risk: Moderate.
	Justification: The study identifies several potential confounders, including gestational age, birth weight, and mode of delivery (vaginal vs. cesarean). Multivariable analysis was conducted to adjust for these factors, with vaginal delivery identified as an independent risk factor for neonatal sepsis. However, the study does not detail all possible confounders, such as socioeconomic factors or access to healthcare, which could also influence the results.
Bias in the selection of participants	Risk: Low.
	Justification: Selection criteria were likely clear, minimizing selection bias.
Bias in the classification of exposures	Risk: Low.
	Justification: The classification of exposure is well-defined and based on objective microbiological criteria.
Bias due to deviations from intended exposures	Risk: Low.
	Justification: As an observational study, there are minimal concerns about deviations from intended exposures.
Bias due to missing data	Risk: Moderate.
	Justification: The handling of missing data and the exclusion of certain neonates could influence the results, introducing a risk of bias.
Bias in the measurement of outcomes	Risk: Low.
	Justification: The outcome measurements are likely accurate, but potential variations in laboratory practices across different hospitals are a minor concern.
Bias in the selection of the reported result	Risk: Moderate.
	Justification: There may be selective reporting, particularly in emphasizing certain pathogens like *Burkholderia cepacia*.
Overall risk of bias	The overall risk of bias in this study is likely Moderate. The study provides important insights into neonatal sepsis in Yemen, particularly in the context of emerging antibiotic resistance. However, the lack of detailed adjustment for confounding factors, the handling of missing data, and potential variability in outcome measurement across different hospitals contribute to a moderate risk of bias.
Song et al., 2022	
Bias due to confounding	Risk: Moderate.
	Justification: While the study does consider key factors, it lacks a comprehensive strategy for adjusting confounding variables.
Bias in the selection of participants	Risk: Low.
	Justification: The selection process is well-documented and appropriate for the study’s objectives.
Bias in the classification of exposures	Risk: Low.
	Justification: The classification of exposures appears reliable and follows accepted criteria.
Bias due to deviations from intended exposures	Risk: Low.
	Justification: Deviations from intended exposures are unlikely given the observational design.
Bias due to missing data	Risk: Moderate.
	Justification: The study does not discuss the handling of missing data in detail, which is a common issue in retrospective studies. It is unclear how many cases may have been excluded or how missing data were managed.
Bias in the measurement of outcomes	Risk: Low.
	Justification: While measurement techniques are likely robust, the lack of discussion on consistency over time introduces some uncertainty.
Bias in the selection of the reported result	Risk: Low.
	Justification: The study appears to report all relevant results comprehensively.
Overall risk of bias	The overall risk of bias for this study is likely Moderate. The study provides valuable insights into trends in neonatal sepsis, but the lack of detailed adjustments for confounding factors and handling of missing data are notable limitations.
Jin et al., 2022	
Bias due to confounding	Risk: Moderate.
	Justification: The study acknowledges key factors but lacks a comprehensive approach to adjusting for confounding variables.
Bias in the selection of participants	Risk: Low.
	Justification: Participants were included based on clear and consistent criteria, minimizing selection bias.
Bias in the classification of exposures	Risk: Low.
	Justification: Exposure classification is based on the type of pathogen and the timing of sepsis onset, which are standard and clearly defined in the study.
Bias due to deviations from intended exposures	Risk: Low.
	Justification: The retrospective nature of the study means there are few concerns about deviations from intended exposures.
Bias due to missing data	Risk: Moderate.
	Justification: The study does not extensively discuss missing data, but it excludes cases with incomplete data and potential contamination, which may lead to selection bias. The extent of missing data and its potential impact on results are not fully addressed.
Bias in the measurement of outcomes	Risk: Low.
	Justification: Standardized laboratory procedures were used to measure outcomes, reducing the risk of measurement bias. However, the lack of discussion on consistency over time introduces some uncertainty.
Bias in the selection of the reported result	Risk: Low.
	Justification: The study appears to comprehensively report all relevant findings.
Overall risk of bias	The overall risk of bias in this study is likely Moderate. The study provides valuable insights into neonatal sepsis and antibiotic resistance, but the lack of detailed adjustments for confounding factors and handling of missing data are notable limitations.

Results

Ultimately, eight articles met all inclusion criteria and were selected for in-depth review. These studies collectively provided significant insights into the microbial dynamics and resistance profiles within NICU settings, thereby contributing to the understanding and management of neonatal bloodstream infections.

A previous study by Sana et al. reported on the incidence of bloodstream infections in 640 neonates, of which 209 exhibited positive blood cultures, culminating in a focused study group of 172 neonates. The study revealed a 58% prevalence of late sepsis, primarily caused by a dominant *Klebsiella* strain. Analysis of the bacterial isolates indicated a predominance of gram-negative bacteria (57%), alongside a 14% detection rate of fungal pathogens. Resistance patterns emerged with 10 *Staphylococcus aureus* strains and 32 other staphylococcal strains showing resistance to methicillin and four enterococci strains resistant to vancomycin. Fungal isolates were found to be sensitive to amphotericin B. The study also identified carbapenems, glycopeptides, polymyxins, and linezolid as effective treatments for both gram-positive and gram-negative bacterial infections. Risk factors such as prematurity and low birth weight were statistically significant (p≤0.01), with respective prevalence rates of 78% and 79% [17].

Li et al. studied 3,454 neonates diagnosed with sepsis out of 26,296 admitted to the NICU over a four-year period. A total of 976 neonates had a positive culture, of which 635 were excluded due to incomplete data, with 341 neonates being eventually included in the study. Among these, 161 cases were classified as early sepsis, predominantly associated with premature birth, low birth weight, premature rupture of the membranes, and additional complications necessitating extended hospitalization and antibiotic therapy in comparison with neonates diagnosed with late sepsis. Furthermore, neonates with late sepsis generally were full term, with normal birth weight, and displayed higher appearance, pulse, grimace, activity, and respiration (APGAR) scores at both the first and fifth minutes, compared to those with early sepsis. Symptomatology and laboratory findings also differed markedly between early and late sepsis cases, with early sepsis neonates presenting with conditions such as respiratory distress, jaundice, hypoglycemia, perinatal asphyxia, and pulmonary hypertension, while late sepsis cases often showed symptoms like fever, feeding intolerance, and abdominal distension. As for the laboratory tests, neonates with early sepsis presented with abnormal lymphocyte count, elevated CRP, and thrombocytopenia, whereas late sepsis complicated neonates more often with abnormal lymphocyte count, elevated CRP, and low hemoglobin levels. The microbial analysis highlighted a higher prevalence of gram-positive bacteria, particularly coagulase-negative staphylococci, and among gram-negative isolates, *Escherichia coli*, *Alcaligenes xylosoxidans*, and *Klebsiella pneumoniae* were prevalent. Resistance patterns varied, with gram-positive isolates largely resistant to penicillin but sensitive to vancomycin, linezolid, minocycline, and tigecycline. Gram-negative isolates showed resistance to ampicillin but sensitivity to amikacin and imipenem, while fungi were sensitive to all treatments (flucytosine, amphotericin B, fluconazole, itraconazole, voriconazole). Finally, early infections revealed methicillin-resistant *Staphylococcus aureus* (MRSA) strains, while methicillin-sensitive *Staphylococcus aureus* (MSSA) strains were predominantly found in late infections, constituting 83.33% of the cases [18].

Mintz et al. conducted a decade-long study involving 15,947 neonates admitted to the NICU, identifying 829 with one or more episodes of bacteremia, resulting in a total of 937 episodes given that 81 neonates had multiple episodes of bacteremia. The majority of these cases (n=870) were classified as late onset. Early bacteremia cases were commonly caused by *Escherichia coli* and Group B *Streptococcus* strains, while late bacteremia cases were frequently associated with coagulase-negative staphylococci and *Klebsiella* strains. Regarding antibiotic use, it appeared that gram-positive bacteria were susceptible to vancomycin and, except for coagulase-negative staphylococci strains, to ampicillin. A further analysis of antibiotic susceptibility over time revealed a decrease in resistance to meropenem for gram-negative bacteria, from 11% to 0%, whereas resistance increased for cotrimoxazole and piperacillin from 17.1% to 34.7% and from 39.4% to 68.4%, respectively. This suggests an evolving resistance pattern among pathogens associated with late-onset infections compared to those implicated in early sepsis, which exhibited greater resistance to ampicillin and cephalosporins [19].

Song et al. conducted a retrospective analysis of NICU admissions over a 20-year period, documenting 14,059 neonatal admissions. Among these, bacteremia was confirmed in 741 cases through positive blood cultures accompanied by clinical indicators of sepsis. This study exhibited 876 sepsis episodes affecting 741 neonates, with a total of 895 bacterial isolates identified. The distribution of sepsis episodes was categorized into early and late onset; early sepsis occurred in 52 neonates across 50 neonates, with two neonates experiencing multiple episodes, while late sepsis was identified in 824 cases among 691 neonates, yielding 843 bacterial isolates. Microbiological analysis revealed a predominance of gram-positive bacteria, accounting for 75.3% of isolates, primarily consisting of coagulase-negative staphylococci and *Staphylococcus aureus*. Conversely, gram-negative isolates predominantly included *Klebsiella pneumoniae*, *Enterobacter cloacae*, *Escherichia coli*, *Burkholderia cepacia*, and *Pseudomonas aeruginosa*. Moreover, a longitudinal assessment of antibiotic susceptibility patterns demonstrated an increase in the resistance of *Staphylococcus aureus* to oxacillin over the study period. Similarly, increased susceptibility to gentamicin, cefotaxime, and ceftriaxone was observed among *Klebsiella pneumoniae* and *Enterobacter cloacae* strains. Interestingly, all pathogens maintained sensitivity to vancomycin [20].

Following on, Jin et al. investigated a cohort of 188,070 neonates over an eight-year span, with 49,094 requiring hospitalization within 28 days of birth. Blood cultures were performed on 46,603 neonates after admission, identifying 1,312 positive cases. A total of 864 neonates were enrolled in the study of which 609 had late sepsis. Cultures revealed 670 gram-positive bacterial isolates, predominantly coagulase-negative staphylococci strains (n=552). The gram-negative bacterial spectrum was primarily composed of *Klebsiella pneumoniae*, *Escherichia coli*, and *Enterobacter cloacae* isolates. Antibiotic resistance profiling showed that 70.6% of coagulase-negative staphylococci strains were resistant to oxacillin, yet exhibited 100% susceptibility to linezolid, vancomycin, and tigecycline. In contrast, *Klebsiella pneumoniae* displayed resistance to ertapenem and imipenem, whereas *Escherichia coli* and *Enterobacter cloacae* were susceptible. All fungal isolates tested were susceptible to amphotericin B, 5-fluorocytosine, voriconazole, fluconazole, and itraconazole [21].

In the study by Salah et al., 199 neonates were assessed, among whom 154 developed positive blood cultures; more specifically, 50% of these cases were classified as early sepsis. The study highlighted that neonates delivered vaginally presented a threefold increased risk of sepsis (odds ratio (OR)=3.08, 95% confidence interval (CI): 1.54-6.16; p=0.002). Significant associations with neonatal sepsis included hyperthermia (p=0.045), convulsions (p=0.01), and elevated CRP levels (p=0.001). Except for two cases where fungal infections were identified, all neonates were infected with bacterial strains. From the cultures, 161 pathogens were isolated; 119 of these were gram-negative bacteria, predominantly *Burkholderia cepacia* and *Klebsiella oxytoca*. Among gram-positive bacteria, *Staphylococcus haemolyticus* and *Staphylococcus epidermidis* were the primary isolates. The study noted resistance patterns where the most frequently detected pathogens exhibited resistance to commonly used antibiotics such as ampicillin, although *Klebsiella oxytoca* strains remained susceptible to gentamicin, amikacin, cephalosporins, and carbapenems. Additionally, *Staphylococcus* strains displayed resistance to ciprofloxacin [22].

In addition, Liu et al. reported on 2,752 bacterial and fungal isolates derived from 2,693 neonates, identifying 1,092 pathogens implicated in neonatal disease. Of these, 702 were associated with late-onset hospital-acquired sepsis and 349 with early-onset sepsis. The demographic analysis revealed that neonates with early-onset sepsis were predominantly full term with normal birth weights, whereas those affected by late-onset sepsis were generally premature, exhibiting extremely low to very low birth weights. Microbiologically, gram-negative bacteria were the predominant pathogens. In particular, *Escherichia coli* and Group B *Streptococcus* were the most frequently isolated in early sepsis cases, while *Klebsiella pneumoniae*, *Escherichia coli*, and various fungal strains constituted 12.8% of the isolates prevalent in late sepsis cases. Antibiotic susceptibility testing indicated that *Acinetobacter baumannii* and *Escherichia coli*, both responsible for early sepsis, were generally susceptible to carbapenems, although *Escherichia coli* exhibited resistance to ampicillin and third-generation cephalosporins, with 44.4% demonstrating polyresistance to multiple antibiotics. For late-onset sepsis, particularly among gram-negative isolates, there was a notable resistance to ampicillin, third-generation cephalosporins, and carbapenems. Gram-positive bacteria, including coagulase-negative staphylococci and *Staphylococcus aureus*, displayed methicillin resistance. Conversely, these strains, along with Group B *Streptococcus*, showed susceptibility to vancomycin. Fungal pathogens exhibited broad susceptibility to 5-fluorocytosine, amphotericin B, and voriconazole, although *Candida albicans* strains were resistant to fluconazole and itraconazole [23].

Finally, Oo et al. conducted a three-year period study, exploring 10,935 neonates across two NICUs, with 1,705 being suspected of sepsis. Cultures were obtained from 1,615 neonates, resulting in 672 positive findings, of which 378 were diagnosed with late-onset sepsis. The risk factors identified with a linkage to sepsis in cases with positive cultures were emergency cesarean section (adjusted prevalence ratio (aPR): 1.2 (95% CI: 1.1-1.4, p=0.001)) and absence of perinatal asphyxia (aPR: 1.2 (95% CI: 1.0-1.7, p=0.01)), and late-onset sepsis had an aPR of 1.2 (95% CI: 1.1-1.4, p=0.008). Microbiological findings indicated a predominance of gram-negative bacteria, specifically isolates of *Klebsiella pneumoniae*, *Acinetobacter*, *Serratia marcescens*, and *Enterobacter*. Among gram-positive isolates, coagulase-negative staphylococci were most frequently identified. Antibiotic susceptibility profiles revealed high resistance among gram-positive bacteria to ampicillin, the amoxicillin-clavulanic acid combination, and cefotaxime, including the piperacillin-tazobactam combination. However, these strains demonstrated susceptibility to vancomycin, amikacin, and linezolid. In contrast, gram-negative bacteria exhibited resistance to ceftazidime, combinations of amoxicillin-clavulanic acid, gentamicin, and amikacin [24].

Characteristics and sample sizes (Table 2) as well as an overview of antibiotic susceptibility of various microorganisms in the included studies (Table 3) are presented below.

**Table 2:** Characteristics and sample sizes of the included studies NICU(s): neonatal intensive care unit(s)

Authors and year of publication	Study design	Sample	Sample characteristics
Sana et al., 2019 [17]	Descriptive, cross-sectional	Neonates in NICU	Total: N=640. Positive blood cultures: N=209. Final sample: N=172. Premature: N=134. Late sepsis: N=100
Li et al., 2019 [18]	Retrospective, cohort	Neonates with risk factors or clinical signs of sepsis	Total: N=26,296. Sepsis: N=3,454. Positive blood cultures: N=976. Cases excluded due to incomplete data: N=635. Final sample: N=341. Early sepsis: N=161. Late sepsis: N=180. The most common clinical manifestations in early sepsis were respiratory distress, jaundice, hypoglycemia, pulmonary hypertension, and neonatal asphyxia. The most common clinical manifestations in late sepsis were fever, feeding difficulties, and abdominal distension
Mintz et al., 2020 [19]	Retrospective	All neonates in the NICU with a positive blood or cerebrospinal fluid culture	Total: N=15,947. Αt least one episode of bacteremia: N=829 (5.2%). Multiple episodes of bacteremia: N=81. Total bacteremia episodes=934
Liu et al., 2021 [23]	Retrospective	Neonates in NICU with positive blood culture	Total: N=2,693 from 25 NICUs. Disease-causing pathogens: N=1092. Pathogens caused early sepsis: N=349 (32%). Pathogens caused community-acquired late sepsis: N=41 (3.7%). Pathogens caused hospital-acquired late sepsis: N=702 (64.3%)
Oo et al., 2021 [24]	Cross-sectional	All neonates in the NICU suspected of sepsis	Total: N= 10,935 from two NICUs. Suspected of sepsis: N=1,705. Blood cultures were performed on N=1,615 neonates. Positive blood cultures: N=672. Neonates born with emergency cesarean section were more likely to have bacterial sepsis compared to normal deliveries. Rate of early sepsis: 43.7%. Rate of late sepsis: 56.3%
Salah et al., 2021 [22]	Cross-sectional	Neonates admitted to NICUs of six hospitals with suspected sepsis	Total: N=199. Positive blood cultures: N=154. Early sepsis: N=100
Song et al., 2022 [20]	Retrospective	All neonates admitted to the NICU with a positive blood culture	Total: N=14,059. Cases of bacteremia: N=741. Sepsis episodes: 876. Bacterial isolates: 895. Early sepsis: N=52. Late sepsis: N=691
Jin et al., 2022 [21]	Retrospective	Neonates admitted to the NICU with clinical signs of sepsis and positive blood cultures	Total: N= 46,094. Positive blood cultures: N=1,312. Neonates enrolled: N=864. Early sepsis: N=255. Late sepsis: N=609

**Table 3:** Overview of antibiotic susceptibility of various microorganisms in the included studies spp.: species

Authors and year of publication	Microorganisms	Antibiotic susceptibility
Sana et al., 2019 [17]	Gram (-) (57%): *Klebsiella pneumoniae*, *Acinetobacter baumannii*, *Serratia marcescens*, *Burkholderia cepacia*, *Escherichia coli*, *Stenotrophomonas maltophilia*, *Citrobacter freundii*, *Enterobacter cloacae*	Gram (-): Polymyxin
	Gram (+) (29%): Coagulase-negative staphylococci, methicillin-resistant *Staphylococcus aureus*, *Enterococcus faecium*, *Enterococcus faecalis*, *Staphylococcus aureus*, *Streptococcus pyogenes*, *Streptococcus anginosus*	Gram (+): Clindamycin, teicoplanin, linezolid, vancomycin. *Streptococcus* spp.: Ampicillin, amoxicillin-clavulanic acid, ceftriaxone, ciprofloxacin, erythromycin, penicillin
	Fungi (14%): *Candida parapsilosis*, *Candida albicans*, *Candida glabrata*, *Candida tropicalis*, *Candida krusei*	Fungi: *Candida* spp.: Amphotericin B
Li et al., 2019 [18]	Gram (+) (57.49%): Coagulase-negative staphylococci, *Streptococcus* spp., *Staphylococcus aureus*, *Enterococcus* spp., *Listeria* spp.	Gram (+): Tigecycline, linezolid, vancomycin. Group B *Streptococcus*: Cefotaxime, ceftriaxone, cefuroxime, levofloxacin, moxifloxacin, penicillin. Coagulase-negative staphylococci: Nitrofurantoin, minocycline, rifampicin, teicoplanin. *Staphylococcus aureus*: Ciprofloxacin, nitrofurantoin, gentamicin, levofloxacin, minocycline, moxifloxacin, rifampicin, teicoplanin, sulfamethoxazole-trimethoprim, phosphomycin
	Gram (-) (33.15%): *Escherichia coli*, *Alcaligenes xylosoxidans*, *Klebsiella pneumoniae*, *Pseudomonas* spp., *Enterobacter* spp., *Serratia* spp., *Acinetobacter* spp.	Gram (-): *Alcaligenes xylosoxidans*: Tigecycline, levofloxacin, meropenem, cefoperazone-sulbactam. *Escherichia coli*: Ertapenem, cefmetazole, amoxicillin-clavulanic acid. *Klebsiella* spp.: Amikacin, levofloxacin, phosphomycin, gentamicin
	Fungi (9.36%): *Candida guilliermondii*, *Candida pelliculosa*	Fungi: *Candida* spp.: Flucytosine, amphotericin, fluconazole, itraconazole, voriconazole
Mintz et al., 2020 [19]	Gram (+) (N=612): Coagulase-negative staphylococci, *Enterococcus faecalis*, Group B *Streptococcus*, *Staphylococcus aureus*, *Streptococcus viridans*, *Listeria monocytogenes*, *Bacillus* spp.	Gram (-) and gram (+) (all staphylococci excluded): Amikacin, cefotaxime, ceftazidime, ceftriaxone, ciprofloxacin, clindamycin, cotrimoxazole, gentamicin, imipenem, meropenem, ofloxacin, penicillin, piperacillin-tazobactam
	Gram (-) (N=309): *Klebsiella pneumoniae*, *Escherichia coli*, *Enterobacter* spp., *Serratia* spp., *Pseudomonas*, *Acinetobacter baumannii*, *Citrobacter* spp., *Sphingomonas paucimobilis*, *Proteus mirabilis*, *Stenotrophomonas*	
Liu et al., 2021 [23]	Gram (-) (58.6%): *Klebsiella pneumoniae*, *Escherichia coli*, *Enterobacter* spp., *Serratia marcescens*, *Acinetobacter baumannii*, *Pseudomonas aeruginosa*	Gram (-): *Acinetobacter baumannii* and *Escherichia coli*: Carbapenems
	Gram (+) (33%): Coagulase-negative staphylococci, Group B *Streptococcus*, *Staphylococcus aureus*, *Enterococcus* spp., *Listeria monocytogenes*	Gram (+): Coagulase-negative staphylococci, Group B *Streptococcus*, *Staphylococcus aureus*: Vancomycin
	Fungi (8.4%): *Candida albicans*, *Candida parapsilosis*, *Candida glabrata*, *Candida guilliermondii*, *Candida tropicalis*	Fungi: 5-Fluorocytosine, amphotericin, voriconazole
Oo et al., 2021 [24]	Gram (+) (37.4%): Coagulase-negative staphylococci, *Staphylococcus aureus*, *Enterococcus* spp., *Streptococcus* spp.	Gram (+): Amikacin, vancomycin, linezolid
	Gram (-) (62.6%): *Klebsiella pneumoniae*, *Serratia marcescens*, *Enterobacter* spp., *Burkholderia cepacia*, *Pseudomonas aeruginosa*, *Acinetobacter baumannii*, *Escherichia coli*, *Klebsiella* spp., *Serratia* spp., *Citrobacter* spp., *Acinetobacter* spp., *Coliform* spp., *Pantoea* spp., *Pseudomonas* spp., *Aeromonas* spp., *Proteus mirabilis*, *Kluyvera cryocrescens*, *Elizabethkingia meningoseptica*, *Stenotrophomonas maltophilia*	Gram (-): Levofloxacin, ciprofloxacin, meropenem
Salah et al., 2021 [22]	Gram (+) (26%): *Staphylococcus haemolyticus*, *Staphylococcus epidermidis*, *Staphylococcus hominis*, *Staphylococcus aureus*, *Staphylococcus saprophyticus*, *Enterococcus faecalis*	Gram (+): Moxifloxacin, linezolid, rifampicin. *Staphylococcus* spp.: Ciprofloxacin
	Gram (-) (74%): *Burkholderia cepacia*, *Klebsiella oxytoca*, *Pantoea agglomerans*, *Pseudomonas aeruginosa*, *Klebsiella pneumoniae*, *Pantoea dispersa*, *Acinetobacter baumannii*, *Acinetobacter lwoffii*, *Enterobacter cloacae* complex, *Escherichia coli*, *Achromobacter denitrificans*, *Sphingomonas paucimobilis*	Gram (-): *Burkholderia cepacia*: Cefepime. *Klebsiella oxytoca*: Gentamicin, ciprofloxacin, levofloxacin, tetracycline, nitrofurantoin, trimethoprim-sulfamethoxazole
	Fungi: *Candida albicans*	
Song et al., 2022 [20]	Gram (+) (75.3%): Coagulase-negative staphylococci, *Staphylococcus aureus*, *Enterococcus faecalis*, Group B *Streptococcus agalactiae*	Phase I (1998-2007). Gram (-): *Escherichia coli*: Cefotaxime
		Phase II (2008-2017). Gram (+): *Staphylococcus aureus*: Cefotaxime, oxacillin
	Gram (-) (24.7%): *Klebsiella pneumoniae*, *Enterobacter cloacae*, *Escherichia coli*, *Burkholderia cepacia*, *Pseudomonas aeruginosa*, *Acinetobacter baumannii*, *Serratia marcescens*, *Enterobacter aerogenes*, *Klebsiella oxytoca*	Gram (-): *Klebsiella pneumoniae*: Gentamicin, cefotaxime, ceftriaxone. *Enterobacter cloacae*: Gentamicin, cefotaxime, ceftriaxone
Jin et al., 2022 [21]	Gram (+) (77.5%): Coagulase-negative staphylococci, *Streptococcus agalactiae*, *Enterococcus* spp., *Staphylococcus aureus*, *Listeria monocytogenes*, *Streptococcus pneumoniae*	Gram (+): Coagulase-negative staphylococci: Vancomycin, tigecycline, linezolid, rifampicin, moxifloxacin, tetracycline. *Streptococcus agalactiae*: Vancomycin, linezolid, moxifloxacin, penicillin G, oxacillin, ampicillin. *Enterococcus* spp.: Vancomycin, streptomycin, gentamicin, linezolid. *Staphylococcus aureus*: Vancomycin, linezolid, moxifloxacin, levofloxacin, ciprofloxacin, trimethoprim-sulfamethoxazole, rifampicin, gentamicin
	Gram (-) (19.8%): *Escherichia coli*, *Klebsiella pneumoniae*, *Enterobacter cloacae*, *Serratia marcescens*, *Elizabethkingia meningosepticum*, *Ochrobactrum anthropi*, *Acinetobacter baumannii*, *Rhizobium radiobacter*, *Pseudomonas aeruginosa*, *Sphingomonas paucimobilis*, *Enterobacter aerogenes*, *Klebsiella oxytoca*, *Morganella morganii*, *Citrobacter freundii*	Gram (-): *Escherichia coli*: Amikacin, imipenem, ertapenem, cefotetan, piperacillin-tazobactam. *Klebsiella pneumoniae*: Levofloxacin, ciprofloxacin, tobramycin, amikacin, piperacillin-tazobactam, gentamicin. *Enterobacter cloacae*: Levofloxacin, ciprofloxacin, amikacin, imipenem
	Fungi (2.7%): *Candida* spp.	Fungi: *Candida* spp.: Amphotericin B, 5-fluorocytosine, voriconazole, fluconazole, itraconazole

Discussion

This review synthesizes data from studies conducted in six Asian countries, including Pakistan [17], China [18,21,23], Israel [19], Korea [20], Yemen [22], and Myanmar [24], spanning from 2019 to 2023 and focusing on a total of 6,503 neonates with bloodstream infections in NICUs. The analysis revealed that late-onset sepsis was more prevalent in neonates across the majority of the studies, with the exception of one study conducted in Yemen where early-onset sepsis was more common.

Microbiological profiles from the studies indicated a balanced predominance of gram-positive and gram-negative bacteria. Predominant pathogens identified across the dataset included *Escherichia coli*, *Klebsiella pneumoniae*, coagulase-negative staphylococci, Group B *Streptococcus*, *Acinetobacter*, *Serratia marcescens*, *Staphylococcus aureus*, *Enterobacter cloacae*, and various fungi. Particularly, in four studies, late-onset infections with gram-positive bacteria were more frequent, while only in one study, by Song et al. [20], *Staphylococcus aureus* and coagulase-negative staphylococci strains were predominantly observed. In contrast, the remaining studies primarily reported coagulase-negative staphylococci strains [14,19,21]. These findings are consistent with the results of other studies in terms of the prevalence of pathogens [8,25]. Additionally, three studies conducted in China at different intervals presented varied findings. The earliest study highlighted a predominance of late-onset sepsis caused by gram-positive bacteria [18], the subsequent study reported a predominance of gram-negative bacteria in late-onset sepsis [23], and the latest study again observed a predominance of gram-positive bacteria in late-onset sepsis [21]. These variations underscore the dynamic nature of microbial prevalence in NICU settings across different periods and regions, aligning with broader trends noted in related literature.

The review of antibiotic resistance in four studies revealed observable resistance to antibiotics recommended by the World Health Organization. Specifically, gram-negative bacteria documented in the studies by Li et al. [18] and Liu et al. [23] exhibited resistance to ampicillin. In the study by Oo et al. [24], gram-positive bacteria not only showed resistance to ampicillin but also exhibited tolerance to penicillin. Furthermore, Salah et al. [22] reported resistance to ampicillin and gentamicin across both gram-positive and gram-negative bacterial strains. Regarding antifungal efficacy, *Candida* species demonstrated susceptibility to a range of antifungal agents across the studies. These included amphotericin B [17-19], flucytosine [18], fluconazole [18,19], itraconazole [17,19], and voriconazole [18,19]. However, the study by Liu et al. [23] highlighted a notable resistance to fluconazole and itraconazole among the fungal isolates [19]. This pattern of susceptibility and resistance emphasizes the critical challenge of managing infections effectively within clinical settings, highlighting the need for continuous monitoring and updating of antibiotic stewardship practices.

Antibiotic stewardship refers to the systematic effort to optimize the use of antibiotics to combat microbial resistance, ensure effective treatment of infections, and minimize adverse effects [26]. The review highlighted several instances of antibiotic resistance, particularly to commonly used antibiotics, among both gram-positive and gram-negative bacteria. This resistance trend poses a significant challenge to the management of neonatal sepsis, necessitating robust antibiotic stewardship programs [27]. Implementing antibiotic stewardship in NICUs involves several strategies including rational antibiotic prescribing, regular review and de-escalation of the antibiotic therapy, education and training for healthcare professionals, and, finally, surveillance and reporting of the antibiotic use [28,29]. More explicitly, neonatologists should base antibiotic prescriptions on culture results and local resistance patterns. The variability in pathogen prevalence and resistance, as observed in the included studies, calls for tailored antibiotic protocols that consider the specific microbial landscape of each NICU [18,23,24]. Moreover, a systematic review of antibiotic therapy is crucial. Once culture and sensitivity results are available, there should be a shift from broad-spectrum to narrow-spectrum antibiotics to minimize resistance development [22,30], whereas continuous education programs for healthcare professionals on the principles of antibiotic stewardship and the latest resistance trends are also essential. These ensure that the NICU clinical staff is well-informed and can make evidence-based decisions regarding antibiotic use [30,31]. Lastly, establishing a robust surveillance system to monitor antibiotic use and resistance patterns can provide valuable data for guiding treatment protocols and stewardship interventions [32,33].

Findings from the current review, on the prevalence of pathogens and resistance patterns, highlight also the need for stringent infection control practices to mitigate the spread of resistant bacteria and fungi. Ensuring rigorous hand hygiene practices among NICU staff, caregivers, and visitors is fundamental. Hand hygiene protocols should be strictly enforced and regularly audited with the prospect of reducing the occurrence of infection and infection‐related neonatal death [34]. Furthermore, regular cleaning and disinfection of NICU surfaces and equipment can reduce the risk of pathogen transmission, and therefore, the use of effective disinfectants and adherence to cleaning schedules are of utmost importance [35]. Finally, implementing isolation precautions for neonates infected or colonized with multidrug-resistant organisms can prevent cross-transmission. Cohorting patients and using personal medical equipment, dedicated to each neonate, can also be an effective control measure [36].

## Conclusions

Despite the moderate bias, the studies still contribute valuable insights into the epidemiology and antimicrobial resistance patterns of bloodstream infections in neonates hospitalized in NICUs. However, the findings should be corroborated by further research with more rigorous study designs to confirm the outcomes. In addition, integrating antibiotic stewardship and stringent infection control measures with a robust diagnostic approach, including the use of the neonatal sepsis calculator to accurately estimate the risk of early-onset neonatal sepsis, can significantly reduce unnecessary antibiotic use in non-septic neonates, thereby optimizing treatment outcomes and lessening the development of antibiotic resistance in NICUs.

Late-onset sepsis emerged as the predominant form of infection across the majority of the studies. The microbiological profiles revealed a balanced distribution of gram-positive and gram-negative bacteria. Variations in microbial prevalence were observed across different studies, reflecting the dynamic nature of microbial ecology within NICU settings over time and across regions. Regarding antibiotic resistance, the studies highlighted concerning levels of resistance among both gram-positive and gram-negative bacteria to antibiotics, while fungal isolates, particularly *Candida* species, exhibited susceptibility to various antifungal agents. It should not be disregarded though that the presence of candidemia, according to multiple studies across different regions of the world, is still highly fatal for the neonate and linked to elevated mortality rates. These findings underscore the continuous difficulty of controlling infections in NICU environments and emphasize the necessity for ongoing monitoring, lifelong education and professional development, and the continuous adjustment of antibiotic management strategies to prevent antimicrobial resistance.
